# New Insights for an Advanced Understanding of the Molecular Mechanisms in Oral Squamous Cell Carcinoma

**DOI:** 10.3390/ijms25136964

**Published:** 2024-06-26

**Authors:** Ana Caruntu, Shun-Fa Yang, Julio Acero

**Affiliations:** 1 Department of Oral and Maxillofacial Surgery, Faculty of Dental Medicine, Titu Maiorescu University, 031593 Bucharest, Romania; 2 Department of Oral and Maxillofacial Surgery, Carol Davila Central Military Emergency Hospital, 010825 Bucharest, Romania; 3 Institute of Medicine, Chung Shan Medical University, Taichung 40201, Taiwan; ysf@csmu.edu.tw; 4 Department of Oral and Maxillofacial Surgery, Ramon y Cajal University Hospital, 28034 Madrid, Spain; j-acero@telefonica.net; 5 Department of Oral and Maxillofacial Surgery, Hospital Universitario Puerta de Hierro, 28222 Majadahonda, Spain

Oral squamous cell carcinoma (OSCC), the most common type of head and neck cancer, remains a highly challenging cancer to treat, largely due to the late diagnosis in advanced stages of the disease, which occurs in more than half of cases [1]; however, it is also due to the complex pathogenic mechanisms that impact the response to the currently available therapies [2,3]. Compared to many other malignancies, where recent discoveries—turned into novel treatments—have completely changed the course of life for patients [4,5], in OSCC, the prognosis has not improved significantly in recent decades [6]. In this context, in the medical scientific community, there is a continuous quest to identify novel groundbreaking information that could shed light on the molecular mechanisms that promote OSCC development and offer new avenues for the more effective therapeutic management of this disease.

Genetic and epigenetic changes, alterations in signaling pathways, contribute to the mutational landscape that has been incriminated in the resistance to therapy and impaired prognosis in OSCC [7]. Interventions targeting these types of changes associated with OSCC pathogenesis—TP53 [8], retinoblastoma (RB1) and its protein product [9], epidermal growth factor receptor (EGFR) [10], Cyclin dependent kinase inhibitor 2A (CDKN2A) [11], the MAPK pathway [12], cancer stem cells (CSC) [13], and microRNA (miRNA) [14] expression—have been intensively investigated, sometimes with encouraging results. An example of an already FDA-approved targeted medication, currently under investigation for metastatic OSCC with promising results, is the JAK inhibitor, rituximab [15]. In a recently published study, Huni et al. revealed that the differential expression of microRNA (miRNA) molecules is crucial for chemotherapeutic responsiveness in OSCC [16]. Cells lines of OSCC expressing miR-375 exhibited resistance to all the tested chemotherapeutic agents. At the same time, the resistant cells lines were the only ones to lack the expression of miR-27. These findings reveal important regulatory mechanisms in the development of chemoresistance in OSCC. miRNA profiling could lead to novel biomarkers to predict the treatment response and tailor personalized therapies in different cancers, including OSCC. Another interesting study conducted by Campagna et al. explored the influence of the paraoxonase-2 (PON2) enzyme on the cell viability, proliferation, and sensitivity to chemotherapy in OSCC cell lines [17]. Their findings suggest that PON2 is not merely a bystander but actively contributes to the survival and chemoresistance of cancer cells in OSCC. The modulation of PON2 activity was associated with reduced cell viability and increased sensitivity to cisplatin. Cell lines with a high expression of the PON2 enzyme exhibited cisplatin-resistant features. Further investigation of PON2 could provide novel perspectives for the enhancement of chemotherapy efficacy in OSCC.

Considering that the main prognostic element in OSCC is the moment of diagnosis, significant efforts have been channeled to assess and identify effective diagnostic tools for the early detection of this malignancy [18,19,20]. Salivary proteins that act as chemical barriers and play a defensive role in OSCC were explored in the study conducted by Kalló et al. [21]. They identified alterations in the expression of salivary proteins that could serve as early biomarkers for OSCC, facilitating an early diagnosis and, thus, promoting an improved prognosis for OSCC. The identification of somatic mutations in plasma cell-free DNA is another promising diagnostic approach. Lin et al. identified significant correlations between the plasma mutational burden in cell-free DNA with clinical staging and distant metastatic status in OSCC patients [22]. Using a liquid biopsy technique, they identified the TTN, PLEC, SYNE1, and USH2A genes as being most frequently mutated in OSCC, while RORC, SCL49A3 and NUMBL were significantly associated with metastatic disease. These findings could set the foundation for a minimally invasive method in monitoring the disease progression and tailoring the treatments for OSCC.

Another element that plays a crucial role in OSCC pathogenesis is the tumor microenvironment [23]. The interplay between cancer cells and their surrounding stroma, including immune cells, fibroblasts, and extracellular matrix components, significantly influences tumor growth and the response to treatment. Research also underscores the importance of tumor-infiltrating immune cells in OSCC’s prognosis. In an immunohistochemistry study on specimens of OSCC, Caruntu et al. reported that the density, distribution, and characteristics of the immune cells within the tumor microenvironment significantly affected patient outcomes [24]. The increased infiltration of CD8-positive lymphocytes and CD56-positive cells was independently correlated with the improved survival of patients treated for OSCC. These findings need to be further investigated, because the characteristics of the tumor microenvironment could influence the efficacy of immunotherapy and could set the base for the optimized profiling of patients.

In terms of treatment options for OSCC, the standard of care has been constant in recent decades—surgery, radiotherapy and/or chemotherapy, with the more recent introduction of immunotherapy and targeted therapy for recurrent, metastatic, or advanced disease [25]. Constant attempts have been made to assess different molecules for their anticancer potential in OSCC. In a recently published study, Hsieh et al. investigated the ability of Semilicoisoflavone B, a phenol-based natural compound, to induce cell arrest and apoptosis in OSCC [26]. They reported interferences with multiple signaling pathways associated with OSCC progression such as CDK2A, MAPK, and Ras/Raf/MEK, as well as increased reactive oxygen species (ROS) production. These findings highlight a new avenue for therapeutic intervention in OSCC. Advances in immunotherapy and targeted therapy research offer new horizons of hope for patients with OSCC. Several studies highlight the potential of immune checkpoint inhibitors and novel drug delivery systems designed to enhance the targeting of cancer cells while minimizing the side effects [25,27]. These innovative approaches are crucial for overcoming the limitations of current treatments and achieving better clinical outcomes.

In conclusion, continuing the investigation of the pathogenic mechanisms in OSCC is of paramount importance for improving the survival of patients affected by this devastating disease. Studies conducted in this direction could pave the way for the development of more effective diagnostic tools and therapeutic strategies. It is imperative for the scientific community to build on these findings, fostering collaborations and continued research to ultimately improve the management and prognosis of OSCC.
